# Comparative Analysis of Virulence Factors of Homozygous and Heterozygous Strains of *Candida albicans* Vaginal Isolates

**DOI:** 10.1155/2020/8889224

**Published:** 2020-06-27

**Authors:** Hasti Nouraei, Sahar Sheykhi, Zahra ZareShahrabadi, Hossein Khodadadi, Kamiar Zomorodian, Keyvan Pakshir

**Affiliations:** ^1^Department of Parasitology and Mycology, School of Medicine, Shiraz University of Medical Sciences, 7134845794, Shiraz, Iran; ^2^Basic Sciences in Infectious Diseases Research Center, School of Medicine, Shiraz University of Medical Sciences, 7134845794, Shiraz, Iran

## Abstract

Although the epidemiology of pathogenic *Candida* species is changing due to invasive diseases, *Candida albicans* has become the common cause of human infections worldwide. *Candida albicans* is a diploid yeast with a mostly clonal mode of reproduction and without known complete sexual cycle. This species has two heterozygous and homozygous strains at hyphal wall protein 1 gene locus (*hwp1*). Little is known about virulence factors of these strains. The aim of this study was to evaluate the exoenzyme activity of heterozygous and homozygous *C. albicans* strains. A total of 60 stock *Candida albicans* species isolates, which consisted of 30 homozygous and 30 heterozygous strains, were used for exoenzyme activities. We used egg yolk agar, Sabouraud blood agar, and bovine serum albumin agar for evaluation of phospholipase, hemolysin, and proteinase activity, respectively. Homozygous strains of *Candida albicans* had more phospholipase and proteinase activity than heterozygous strains. However, there were no significant statistical differences between the two strains in the severity of exoenzymes production. Beta hemolysin activity was seen in 100% and 96.7% of the homozygous and heterozygous strains, respectively. The results of this study indicated that both of the strains exhibited exoenzyme activities in different ranges. There were no significant statistical differences in virulence factors between the homozygous and heterozygous strains.

## 1. Introduction

*Candida albicans* is a commensal diploid organism and inhabits a variety of niches in human populations. It becomes an opportunistic pathogen in immunocompromised patients and can cause a wide variety of infections, ranging from superficial to disseminated infections. *Candida albicans* is the common fungal pathogen and has advanced a range of assumed virulence factors that allow successful colonization and infection of the host under suitable predisposing conditions [1].

*Candida albicans* secretes hydrolytic enzymes, such as lipases, proteases, phospholipases, and hemolysin, which are considered to be integral to their virulence and pathogenesis. Expression of several genes, including *ALS1*, *ALS3*, *ECE1*, and *HWP1*, is required for systemic candidiasis [2]. In particular, the gene hyphal wall protein 1 (*HWP1*) is known to encode a major *C. albicans* protein involved in several functions, including assemblage of cell wall, intracellular signaling, and hyphal expansion by cross-linking to the glucans of cell wall [3, 4]. Moreover, *hwp1* promotes binding of *Candida* to epithelial cells, as the initial step of colonization and causes virulence in systemic candidiasis [4]. Genetics of *C. albicans* as a diploid fungal pathogen has been studied by many researchers [5, 6]. *C. albicans* has two homozygous and heterozygous strains at the *hwp1* locus. One of the methods for the discrimination of these two strains is the amplification of this gene, whose homozygous strains produce one DNA fragment at 941 base pair (bp) while heterozygous strains produce two fragments in either 941 or 839 bp [7–10]. One of the most common kinds of candidiasis in women is *Candida* vaginitis and *Candida albicans* which is reported as the most causative agent [7, 11]. It occurs when *Candida* superficially penetrates into the mucosal layer of the vagina and causes an inflammatory response. The severity of signs and symptoms is typically different in patients [11], and secretion of enzymes as virulence factor is responsible for the severity of symptoms in patients suffering from *Candida* vaginitis [12].

According to Mucci et al. [8], in comparison with symptomatic and asymptomatic patients, there is no significant difference in the prevalence rate of homozygous and heterozygous *C. albicans* strain isolates.

Since the distribution of homozygous and heterozygous strains of *C. albicans* almost exists in the vaginal area, knowing the severity of enzyme secretion in these strains as a virulence factor might be effective in clinical manifestation of patients. The aim of this study was the comparative analysis of phospholipase, proteinase, and hemolysin activity among the homozygous and heterozygous strains of *Candida* vaginitis isolates.

## 2. Materials and Methods

### 2.1. *Candida albicans* Strain Isolates

In this study, a total of 60 stocks of *Candida albicans* species consisting of 30 homozygous and 30 heterozygous strains, which were previously recovered from *Candida* vaginitis patients (ethical code: IR.SUMS.REC.1397.380), were examined. These two strains were identified as homozygous and heterozygous strains level by molecular method previously [13] and kept in −20°C condition. The isolates were subcultured on Sabouraud dextrose agar (Merck, Germany) before using.

### 2.2. Proteinase Secretion Activity

The proteinase secretion was detected by bovine serum albumin medium (BSA) according to Dagdeviren et al. [14]. Six-millimeter filter paper discs were dipped into a suspension of strains with a density of approximately 10^7^ yeast.mL^−1^ and applied to the BSA medium. The plates were incubated at 30°C for 6 days. After incubation, the diameter of opaque zones around the discs was considered as proteinase production. Precipitation zone (Pz) was measured and expressed as negative (−) for no clearance, positive (+) for mild, double positive (++) for moderate, triple positive (+++) for strong, and four positives (++++) for very strong enzyme activity. The standard strain of *C. albicans* (ATCC10261) was used as a positive control.

### 2.3. Phospholipase Secretion Activity

The plate method was used for the estimation of phospholipase activity according to Price et al. [15]. The medium was prepared by adding 5.5 g CaCl_2_ and 58.4 g NaCl to Sabouraud dextrose agar (Merck, Germany). Then sterile egg yolk was centrifuged at 5,000 ×g for 30 min, followed by adding 20 mL of supernatant to the cooled medium. Two McFarland turbidity yeast suspension was prepared; 10 *μ*L yeast suspension was spot-inoculated on the plate medium and incubated at 37°C for up to 5 days. A precipitate zone around the colonies was measured, and precipitation zone was expressed as Pz value from 1 to 4 as follows: Pz 1 (negative), Pz 1+ (0.9–1), Pz 2+ (0.89–0.80), Pz 3+ (0.79–0.70), and Pz 4+ (≥0.69). The standard strain of *C. albicans* (ATCC10261) was used as a positive control.

### 2.4. Hemolysin Activity

Blood plate assay was used for hemolytic activity as described by Yigit and Aktas [16]. Sheep blood Sabouraud dextrose agar was prepared by adding 7% v/v of fresh sheep blood and 3% w/v of glucose. Next, 2 McFarland turbidity suspension was prepared. Then, 10 microliters of inoculation was spotted on sheep blood SDA plates and incubated at 37°C for 48 hours. A ring of lysis around the colonies was considered for hemolytic activity: complete (beta) in case of totally translucent ring, incomplete (alpha) in case of greenish-black halo, or no hemolysis (gamma or none). *Candida albicans* ATCC10261 was used as a positive control.

### 2.5. Statistical Analysis

Results were analyzed using the SPSS (Statistical Package for the Social Sciences) program. Fisher exact test *p* value <0.05 was considered statistically significant.

## 3. Results

### 3.1. Results of Homozygous Strain Tests

Phospholipase activity was seen in 83.3% of the strains with Pz = 0.59 (0.38–1). Also, 66.7% of the strains were strong phospholipase producers.

**Figure 1:**
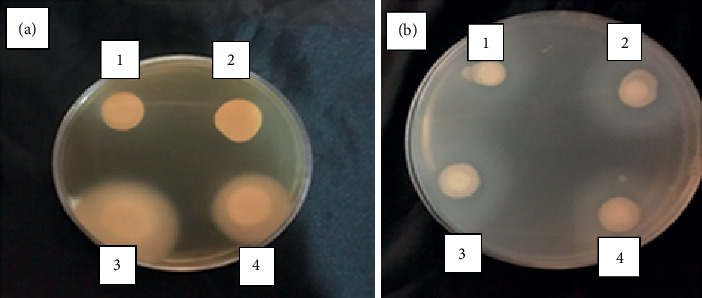
Phospholipase (a) and proteinase (b) activity. (a) Homozygous strain: spots 1, 3 (neg-pos); heterozygous strain: spots 2, 4 (neg-pos). (b) Homozygous and heterozygous strains: discs 1–4 (positive).

All the strains were strong proteinase producers (100%) with Pz = 0.45(0.2–0.85) (Figure 1).

Hemolysin activity was seen in 100% of the strains considering the beta hemolysis pattern. More details are available in Table 1.

### 3.2. Results of Heterozygous Strain Tests

It was observed that 96% of the strains were phospholipase producers with Pz = 0.63(0.3–1) and 76.7% were strong producers (Figure 1).

Proteinase activity was seen in 100% of the strains with Pz = 0.46 (0.3–0.6), and 90% of the strains were strong producers.

All of the strains had hemolysin activity (100%), and beta hemolysis pattern was seen in 96.7% of the strains. More details are presented in Table 1.

### 3.3. Results of Statistical Analysis

Despite the variety of the test results in the two groups, statistical analysis using Fisher exact test reveals no correlation between the two strains in phospholipase activity (*p*=0.29), proteinase activity (*p*=0.23), and hemolysin activity (*p*=1).

## 4. Discussion

Recently, fungal infections have represented a serious public health problem that needs urgent attention as they cause more than 1.5 million deaths worldwide each year. *Candida albicans* is one of the most common reasons of *Candida* infections that can cause both superficial and disseminated infections. *Candida dubliniensis* and *C. africana* are other two yeast species that are closely related to *C. albicans*, which are named as *C. albicans* species complex [7, 17]. One of the best methods for discrimination of these species is the amplification of *hwp1* gene. By this method, three different sizes of DNA fragments that help identify these species are produced [12, 18]. There are many studies about the role of *hwp1* gene in *Candida* pathogenesis and morphogenesis as an important risk factor. Tsuchimori et al. [19] investigated the role of this gene as a virulence factor and reported a reduction in virulence during exposing a homozygous *hwp1* null mutant of *C. albicans* in infected mice. Moreover, homozygous and heterozygous strains of *C. albicans* could be discriminated from each other during amplification of *hwp1* locus by producing one or two DNA fragment sizes [8].

*Candida* vaginitis, as one of the most common *Candida* infections in women, is caused by many species of *Candida,* especially *C. albicans*. *Candida* vaginitis is strongly influenced by multiple factors such as hormone levels, type of organism, physiological condition, diabetes, antibiotic usage, and commensal bacteria. The secretion of extracellular hydrolytic enzymes, biofilm formation, phenotypic switching, adherence to host tissue, and many other factors have been listed as virulence factors and proposed to be involved in pathogenicity of *Candida* species [20, 21]. According to a study by Mucci et al. [8], both homozygous and heterozygous strains were isolated from women with vaginitis. If the secreted exoenzymes had been different between these two strains, this could probably have been responsible for the severity of symptoms in vaginal candidiasis patients. To our knowledge, there is no data about the evaluation of exoenzymes as virulence factors in these strains. The result of our data profile in phospholipase activity demonstrated that 66.7% of homozygous and 76.78% of heterozygous strains were high enzymatic activity producers, with no significant statistical difference between the two strains in the secretion of this enzyme.

Aspartyl proteinase could contribute to tissue damage during vaginal candidiasis infection while the other enzymes caused different tissue damage as well [22]. Regarding proteinase activity, all of the homozygous and heterozygous strains produced a high level of this exoenzyme, and these results indicate that proteinase in both strains can be considered as a virulence factor in the development of symptoms. Our results revealed the fact that both strains have the ability to secrete extracellular enzymes in a different range of severity.

Hemolysin activity was almost similar in the two strains, with heterozygous strain showing lower activity than the other strain. According to our study that showed no significant correlation between pathogenicity mechanisms of these strains and with this huge variability in causing of *Candida* vaginitis, it is necessary to consider other conditions of clinical symptoms in patients.

Reducing symptoms in vaginal candidiasis is spotted as treatment. There are several oral and topical antifungals and some other supplementary components such as phytol (acyclic diterpene alcohol) and avarol (marine natural product) used for this purpose [23, 24]. Centers of Disease Control and Prevention (CDC) recommended oral fluconazole as first-line therapy for vaginal candidiasis. On the other hand, topical imidazoles (i.e., econazole, clotrimazole, miconazole, and ketoconazole) are used and have noticeable effect [25]. Combination therapy of this conventional azole therapy and this supplementary agent may have a synergistic effect in treatment of vaginal candidiasis and could reduce symptoms [26].

Despite the statistical analysis results on exoenzyme activity, which showed no correlation between the two strains, we believe that in the case of increasing the number of samples, the statistical data could be statistically significant.

## 5. Conclusion

In this study, comparative analysis of virulence factors demonstrated that both of the strains could express exoenzymes as virulence factor in different ranges. There were no significant differences in virulence factors and pathogenesis of the two strains. Therefore, according to our data, we could not suggest any relation between the severity of symptoms in vaginal candidiasis patients and the type of these strains. However, more samples need to be examined to confirm our data.

## Figures and Tables

**Table 1:** Comparison of virulence factors in heterozygous and homozygous strains of vaginal *Candida albicans* species.

Strains	Virulence factors					Total
	Phospholipase/Pz mean(range)	Proteinase/Pz mean(range)	Hemolysin (%)			
			*α*	*β*	*γ*	
Homozygous	0.59 (0.38–1)	0.45 (0.2–0.8)	—	100	—	30
Heterozygous	0.63 (0.3–1)	0.46 (0.3–0.6)	—	96.7	3.3	30

## Data Availability

The data used to support the findings of this study were supplied by the Deputy of Research and Technology of Shiraz University of Medical Sciences under license and so cannot be made freely available. Requests for access to these data should be sent to Keyvan Pakshir (pakshirk@gmail.com).
